# Morbidity and mortality meetings to improve patient safety: a survey of 109 consultant surgeons in London, United Kingdom

**DOI:** 10.1186/s13037-019-0207-3

**Published:** 2019-08-19

**Authors:** Daniel M. Sinitsky, Siri B. Gowda, Khaled Dawas, Bimbi S. Fernando

**Affiliations:** 1 Department of Surgery, University College London Hospitals NHS Foundation Trust, 235 Euston Road, London, NW1 2BU UK; 2 University Department of Surgery, Royal Free London NHS Foundation Trust, Pond Street, London, NW3 2QG UK

**Keywords:** Patient safety, Medical error, Clinical governance

## Abstract

**Background:**

Morbidity & Mortality (M&M) meetings are a critical component of clinical governance. They have the potential to improve patient outcomes, quality of care, attitudes towards patient safety and they contribute to the education of clinical staff. This study aimed to evaluate individual surgeons’ experience of these meetings, and to explore their perceived usefulness and barriers to open discussion of adverse outcomes.

**Methods:**

Consultant general surgeons in London, United Kingdom, were invited to anonymously complete an online survey consisting of 18 key items.

**Results:**

Invitations were sent to 323 consultant surgeons from 19 NHS Trusts. Responses were received from 109 (33.7%), of which 99 (90.8%) answered all key items. Seventy-two of 104 (69.2%) attend almost all or all M&M meetings. These were rated as being more conducive for learning than for service improvement (*p* = 0.001). On a scale of 1 to 10 (10 = fearless), 41 of 105 (39.0%) rated as ≤5 the fearfulness of legal or other negative repercussions resulting from open discussion of complications/mortalities. Ninety-eight respondents gave a median rating of 10 (IQR: 8–10) for willingness to talk openly about their complications/mortalities (10 = willing/able).

**Conclusions:**

Many surgeons in London do not routinely attend M&M meetings, despite these occurring within ‘protected time’. There may be a willingness to talk openly about complications, though there exists a fear of litigation. The nature, content and learning potential of such open M&M discussions should be explored in future research.

## Background

The Morbidity & Mortality (M&M) meeting is a forum where adverse outcomes can be discussed. They have the potential to improve patient outcomes [1], quality of care, attitudes towards patient safety [2] and they contribute to the education of clinical staff [3]. They are a mandatory requirement in the United States [4] and should also be attended by all UK surgeons according to Good Surgical Practice [5].

The importance of M&M meetings has been periodically reaffirmed by the publication of various high-profile public enquiries into poor post-operative outcomes [6, 7]. M&M meetings are deemed an important component of clinical governance that provide both the necessary administrative assurances that poor outcomes are being monitored and addressed [8], and the environment in which learning from them may take place. Such learning may occur on an individual level, where M&M meetings theoretically allow open discussion of aspects such as decision-making and technical aspects of surgery. Discussion may also facilitate the recognition of systems and processes that are weak in order to improve outcomes for future patients.

The Royal College of Surgeons (RCS) have emphasized the central role that surgical M&M meetings have in achieving and maintaining high standards of care, and have therefore published guidance to help surgical departments across the UK carry them out effectively and efficiently. This guidance covers aspects that include coordination and administrative support, scheduling frequency and duration, participation and attendees, the identification and presentation of cases for discussion, chairing of meetings and team behaviours, case grading and formal recording [9].

The aim of this study was to evaluate consultant surgeons’ experience of M&M meetings against the RCS guidance, and to conduct an initial exploration of their perceived usefulness and the barriers to open discussion of adverse surgical outcomes.

## Methods

An online cross-sectional survey was designed using QuestionPro (https://www.questionpro.com; San Francisco, CA, USA), with 18 key items (Table 1) utilizing a combination of open and closed questions. It was tested and modified according to feedback from local surgeons, achieving consensus agreement prior to commencement of data collection.

**Table 1:** Survey Design. An optional 19th item was also included, inviting the participant to leave an email address solely to avoid receiving reminder emails

1	How often does your surgical department hold Morbidity & Mortality (M&M) meetings?
	≧ 2x per month / Every month / Every 2 months / Every 3 months / Every 4–6 months / 2x per year / 1x per year / < 1x per year / Never / I don’t know / Other ____________
2	At the time of completing this survey, are you aware of the inclusion criteria for case discussion at your departmental M&M meeting?
	Yes / No *(Question logic: Moves to Q4 if answers No)*
3	What are these inclusion criteria? *(Open question)*
4	Does your M&M meeting include data on outpatient events? This refers to morbidity/mortality that occurs or is identified in the outpatient setting.
	Yes / No / I don’t know
5	Are these M&M meetings within ‘protected time’ (i.e. with no concurrent scheduled elective activities)?
	Yes / No / I don’t know
6	Please estimate the proportion of scheduled M&M meetings that you have attended in the last 12 months.
	None / Rarely / Less than a quarter / Less than half / Around half / More than half / Almost all / All
7	Who routinely attends your department’s M&M meetings? Select all that apply.
	Medical Students / FY1 Doctors / SHOs (including FY2) / Registrars / Other clinical specialties / Nursing staff (at least one) / Managerial staff (at least one) / Other (please state)
8	In your department’s M&M meetings, is there routinely a clearly-delegated chair person?
	Yes - consultant surgeon / Yes – non-consultant grade / No / I don’t know
9	How judgmental do you find the environment within the M&M meeting? (1 = very judgmental, 10 = non-judgmental)
10	How would you rate your own willingness or ability to talk openly about your complications/mortalities during the M&M meeting? (1 = unwilling/unable, 10 = willing/able)
11	Please rate your fear of criticism from your peers during M&M meetings (1 = very fearful, 10 = fearless)
12	Please rate your fear of legal or other negative repercussions resulting from completely open discussion of your complications/mortalities (1 = very fearful, 10 = fearless)
13	Are there any other factors that hinder your openness in discussion of your complications during an M&M meeting? *(Open question)*
14	How conducive do you feel your M&M meetings are for learning? (1 = not at all, 10 = highly conducive)
15	How conducive do you feel your M&M meetings are for service improvement? (1 = not at all, 10 = highly conducive)
16	To what extent do you feel individuals’ performance (e.g. decision-making) receives the focus of M&M discussions? (1 = not at all, 10 = exclusively about individuals’ performance)
17	To what extent do you feel systems and processes (e.g. equipment issues, staffing levels, pathway deficiencies) receive the focus of M&M discussions? (1 = not at all, 10 = exclusively about systems and processes)
18	How are the discussions/outcomes disseminated following an M&M meeting?
	I don’t know / M&M meeting records are not available / They are given or sent to me in paper format / They are emailed to me / They are accessible but I do not know how to obtain them / They are accessible and I know how to obtain them / Other (please state)

Between October and December 2018, invitations to complete the survey anonymously were sent by email to consultant general surgeons practicing in London, United Kingdom (defined as the area bound by the M25 motorway). An optional 19th item was included at survey completion, allowing responders to leave their email address for the sole purpose of avoiding any future reminder email, of which three rounds were otherwise sent.

Data was downloaded onto an Excel spreadsheet (Microsoft, Redmond, WA, USA) for descriptive analysis. Using IBM SPSS Statistics version 25 (Armonk, NY, USA), data normality was tested using the Shapiro-Wilk test, and then the Wilcoxon matched-pair signed-rank test was used to compare medians between items 11 and 12 (paired ratings for fear of criticism versus legal repercussions during M&M meetings), items 14 and 15 (conduciveness of M&M meetings for learning versus service improvement), and items 16 and 17 (focus of M&M discussions on individuals versus systems and processes). Results were considered statistically significant where *p* < 0.05.

## Results

**Fig. 1:**
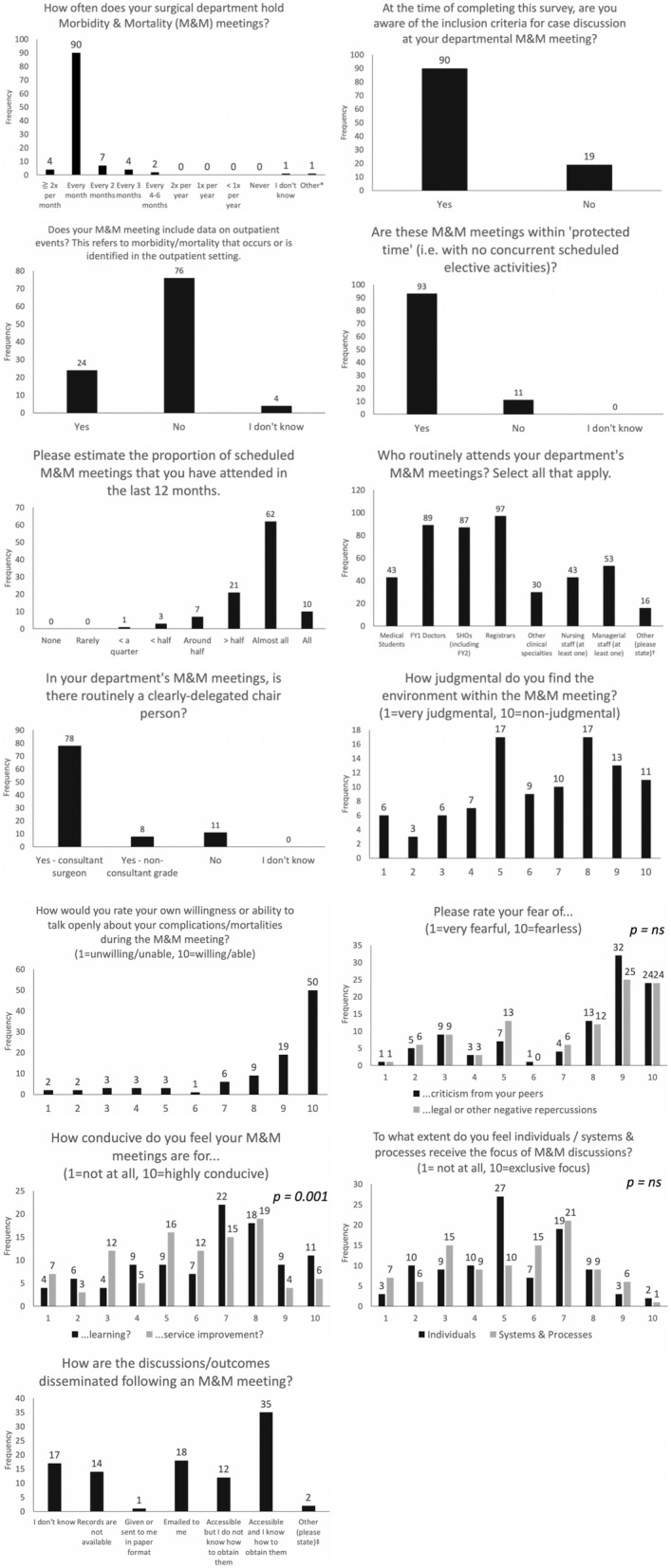
Morbidity & Mortality Meeting Survey Results. *“Mortality reviews weekly. M&M monthly;” †14 responded “consultants”, one with “consultants, associate specialists” and one with “variables e.g. ward or theatre matron when relevant;” ‡“Action logs visited at subsequent meetings” and “Good question! I know they are recorded, I used to get them regularly as email, but not seen outcome for the last 5 months.” ns = not significant

Email invitations were sent to 323 consultant general surgeons across 19 NHS Trusts (mean 17.0 consultants per Trust). Eight of these Trusts cover major teaching hospitals. There were 109 (33.7%) survey responses, of which 99 (90.8%) had responded to all 18 key items. Results are displayed in Fig. 1.

Ninety-four of 109 (86.2%) of responders indicated that their department holds M&M meetings once or twice per month. Ninety of 109 (82.6%) were aware of the inclusion criteria for case discussion. When asked to enter these criteria, 10 of 47 (21.3%) used the Clavien-Dindo classification system for surgical complications, the remainder were non-specific and variable in their description, other than for mortality. Seventy-six of 104 (73.1%) respondents indicated that their department does not include data from outpatient events in their discussion. Ninety-three of 104 (89.4%) indicated that their department held M&M meetings within ‘protected time’ (i.e. no concurrent scheduled elective activities). Of 104 respondents, 11 (10.6%) indicated an attendance of around or less than half of M&M meetings within the previous 12 months, with 21 (20.2%) indicating attendance of more than half and 72 (69.2%) indicating their attendance at ‘almost all’ or ‘all of them’.

When asked who routinely attends M&M meetings, of 102 responders notably 89 (87.3%) indicated medical student attendance, 43 (42.2%) indicated nursing staff, and 53 (52.0%) indicated routine attendance by managerial staff. Seventy-eight of 97 (80.4%) respondents indicated that there was a clearly-delegated consultant-grade chair person, with 11 (11.3%) indicating that there was no clearly-delegated chair person.

Of 99 respondents, 39 (39.4%) rated the judgmentalism of M&M meetings as 5 or less, and the overall median rating was 7 (IQR: 5–8), where 1 = very judgmental and 10 = non-judgmental. Ninety-eight respondents gave a median rating of 10 (IQR: 8–10) for their individual willingness to talk openly about their own complications/mortalities, where 1 = unwilling/unable and 10 = willing/able. Seventeen of 99 (17.2%) do not know how outcomes are disseminated following M&M meetings and 14 of 95 (14.1%) indicate that records are not available.

The difference between the median ratings for the conduciveness of M&M meetings to learning (7, IQR: 5–8) and service improvement (6, 4–8) was statistically significant (*p* = 0.001), where 1 = not conducive at all and 10 = highly conducive. However, there was no significant difference between the median ratings for the fear of criticism (9, IQR: 5–9) and legal repercussions (8, IQR 5–9) as barriers to open discussion of surgical complications/mortalities (*p* = 0.274), where 1 = very fearful and 10 = fearless. Forty-one of 105 (39.0%) rated the fear of litigation as 5 or less. No significant difference existed between the median ratings for the focus of discussions on individuals (5, IQR: 4–7) and systems and processes (6, IQR: 3–7; *p* = 0.824), where 1 = not at all and 10 = exclusive focus.

Finally, when asked to specify any other factors that hinder openness of discussion of complications at M&M meetings, 16 of 35 (45.7%) responded ‘no’ or ‘none’. The remaining 19 (54.3%) responses are listed in Table 2.

**Table 2:** Q13: Are there any other factors that hinder your openness in discussion of your complications during an M&M meeting?

“Blame culture and biasedness.”	
“Changeover of junior staff.”	
“Cross site work.”	
“Dominant personalities.”	
“I am very open and transparent clinician.”	
“I fear people think I’m a useless surgeon - I have high complications because I look after all the emergency patients.”	
“If there is an ongoing investigation about it. ”	
“It’s not an open and honest meeting.”	
“Non-productive discussions. Criticism or showing off .... counterproductive meetings.”	
“Not really but am a senior consultant. Much more difficult for non-consultants to participate.”	
“Occasionally, some factors are more appropriate to discuss with the head of department/other consultants due to their sensitivity.”	
“Personal vendettas.”	
“Protecting other clinicians involved.”	
“Some individuals unfortunately still use these meetings to settle personal griefs and settle scores and get away with it so often. There still seems to be a rule for some and a different one for certain others.”	
“Sometimes the meeting is too soon after the event to have all the relevant information available.”	
“The judgemental attitude. The fact that some people put up all their complications, others you know have happened but they never get discussed. And the lack of defined outcome.”	
“There are different rules for different people.”	
“Time. Our meetings are not frequent enough so we often don’t have as much time as we would like.”	
“Yes, the fact that a member of management attends. It should only be doctors.”	

## Discussions

The results from this London-wide survey indicate that there is substantial room for improvement in the number of surgeons routinely attending M&M meetings even though a large majority suggested that they are within ‘protected’ time. Despite the RCS recommendation that non-clinical managers should be invited [9], only half of respondents indicated their routine attendance. Since there appears to be substantial and fairly equal focus between individuals and systems and processes, their presence might seem ideal given their position to affect change in the latter. Furthermore, this result is despite a view that organisational factors should ideally be more of a focus than individuals during incident reviews [10, 11].

The results of the present study also indicate substantial room for improvement in the way outcomes of M&M discussions are disseminated. They also suggest that consultant surgeons perceive that M&M discussions are focused significantly more on learning rather than on service improvement. Indeed, the view that these meetings are largely educational are shared by medical and surgical residents too [4, 12].

Arguably the most interesting result in this survey was the widespread reported willingness for surgeons to talk openly regarding their own complications. Given the list of compelling inhibitory factors reported by a substantial number of survey respondents, it is possible that the apparent willingness to discuss complications openly may actually reflect a bias to report ideals and perceived best practice rather than on reality.

On face value, the results would be encouraging as clinicians are increasingly being encouraged to learn from error rather than apportion blame [13, 14], despite cognisance of the threat of criminal prosecution [15]. However, this survey does not distinguish between discussion of cases where complications have occurred due to the nature of the pathology, and those that have occurred due to error. *What* is discussed has not been explored, nor the depths of reflection and root cause analysis, but simply whether or not surgeons feel willing and able to discuss openly. The shock and denial that follows a medical error [16] may preclude its recognition, such that effective discussion at M&M meetings could not take place in these scenarios.

The recent high-profile conviction of paediatrician, Dr. Bawa-Garba, for gross negligence manslaughter following the tragic death of a 6-year old boy, may contribute to the growing fear of litigation [17]. Indeed, the present study has found that a large proportion of surgeons are fearful from ‘legal or other negative repercussions resulting from completely open discussion of complications/mortalities’. In the Bawa-Garba case, the written notes of Dr. Bawa-Garba’s clinical supervisor, a prosecution witness, that recorded Dr. Bawa-Garba’s reflections were shared with the hospital investigation and made available to the prosecuting QC [18]. Moreover, according to the General Medical Council, there are no legal protections in the United Kingdom that prevent the use of a doctor’s own written reflections in the course of litigation [19]. This may send a strong signal that undermines the critical patient safety agenda that encourages learning from errors. Therefore, it may be important to explore the content and nature of discussions during M&M meetings in future research, and whether or not such barriers to openness exist in other jurisdictions of disparate legal climates and protections.

Doubts exist as to the effectiveness of M&M meetings in improving outcomes, and this has been attributed, for example, to the use of non-standard approaches, the absence of relevant staff and the under-utilisation of accepted models for incident analysis [20]. However, standardization of case selection and structured presentations can increase participation, educational value and the perception of an increased impact on future patient care [21, 22]. Refocusing M&M meetings on Quality Improvement (QI) education can transform them into a source of QI projects and help to ‘close the loop’ [23, 24], deemed crucial objectives of the modern M&M meeting [25], while satisfying the ACGME’s recent ‘Common Program Requirement’ for QI education in the United States [26].

The focus on QI within an M&M meeting may improve patient safety culture within an institution [2]. Hoffman et al introduced ‘quality minute’ slides on QI to their M&M meetings and observed increases in patient safety incident (PSI) reporting following each presentation [27]. In Leicester, UK, one group introduced seven ‘enhanced’ M&M meetings and orthopaedic specialty trainees were surveyed, with post-intervention results from a safety cultural assessment tool suggesting that such meetings have the potential to positively impact on patient safety knowledge, awareness and attitudes [13]. Such cultural shift may translate into safer care: the group in Leicester subsequently observed a reduction in PSIs [28], and Birkmeyer et al revealed a significant association between safety culture and PSIs among 22 hospitals in Michigan [29].

To the authors’ knowledge, the present study is the only survey in the UK to explore practice and perceptions by consultant surgeons of M&M meetings. It must be emphasized that the results do not reflect the proportion of surgical departments that adhere to any particular guidance criteria, but only the experience of individual surgeons, many of whom will participate in the same meetings.

Limitations of this study include the relatively low response rate of 34%. While this may present a risk of response bias, the authors do not believe this to be significant. Although it is felt that the absolute number of responses received allowed for a reasonable spread of experience across London’s many hospitals, this spread cannot be guaranteed, since requesting the names of Trusts or individuals in the survey may have introduced significant bias. Finally, in retrospect, it is possible that capturing demographic data such as ‘years of experience’, and the extent to which M&M meetings are standardized and focused on QI may have allowed a more in-depth analysis.

## Conclusions

A sizeable number of surgeons in London do not routinely participate in regular M&M meetings. They report a willingness to talk openly about their complications, though there is a substantial proportion that may feel inhibited by fear of legal or other negative repercussions. Surgeons also feel M&M discussions focus more on learning than service improvement. Future study should attempt to explore the barriers to full attendance by surgeons at M&M meetings, the content of M&M discussions, and establish if and how M&M discussions facilitate positive change and better standards of care.

## Data Availability

On request.

## Abbreviations

ACGME: Accreditation Council for Graduate Medical Education
IQR: Interquartile Range
M&M: Morbidity and Mortality
PSI: Patient Safety Incident
QI: Quality Improvement
RCS: Royal College of Surgeons
